# In safe hands: a qualitative study on older adults’ experiences of a tailored primary health care unit

**DOI:** 10.1080/02813432.2022.2097611

**Published:** 2022-07-15

**Authors:** Ulrika Westerling, Mikko Hellgren, Liselotte Hermansson, Emma Nilsing Strid

**Affiliations:** a University Health Care Research Center, Faculty of Medicine and Health, Örebro University, Örebro, Sweden; b Kumla Primary Health Care Center, Faculty of Medicine and Health, Örebro University, Örebro, Sweden

**Keywords:** Primary health care, qualitative research, content analysis, health services for the aged, geriatrics, patient satisfaction

## Abstract

**Objective:** Today’s health care system faces challenges in meeting the needs of older people with multimorbidity. To better cope with these needs, tailored primary health care with geriatric competence and person-centred care has been suggested. The aim of this study was to explore older patients’ experiences of a tailored primary health care unit.

**Design:** This was a qualitative study using semi-structured individual interviews and qualitative content analysis.

**Setting and patients:** Nineteen patients were recruited from a tailored PHC unit for people aged 75 years or older in a region in central Sweden.

**Methods:** The interview data were analysed using inductive category development.

**Results:** In the analysis, the theme *In safe hands when in need of primary health care* emerged. The interviewees expressed a desire to participate in their own care. Easy access, enough consultation time and a calm environment, along with the PHC professionals’ welcoming and attentive approach enhanced their feeling of being in safe hands. PHC professionals were perceived as having geriatric knowledge and taking responsibility for the care of older patients. Although the interviewees experienced that they received attention for their health conditions, a need for a more preventive approach to care emerged.

**Conclusion:** Older patients highly appreciated their tailored PHC unit and they emphasised that it was an improvement compared to the ordinary PHC centre. This study provides insights into older patients’ experiences, which may be helpful in the ongoing process of improving care for older patients in PHC.

**KEY POINTS**
- Older patients attending a tailored Primary health care (PHC) unit felt acknowledged, unlike in the ordinary PHC centre, which facilitated their participation in their care.
- The calm environment, specialist geriatric competence and ample patient contact time enabled them to feel secure and taken care of.
- Older patients expressed a need for an incorporation of social services and health promotion visits at the tailored PHC unit.

## Introduction

Lifespans have lengthened globally over recent decades, with a corresponding increase in the number of people living with chronic conditions. Older people with multiple morbidity and complex needs often have a high degree of disability, large consumption of care and increased mortality [1]. This poses a great challenge for health care systems. The care of older people has been described as fragmented, illness-centred and suffering from a lack of competencies [2]. A transition from hospital-based care to outpatient and preventive care in the primary health care (PHC) systems is anticipated [3,4]. There is a general consensus that a more holistic view of patients with complex needs is important for the quality of their health care [3,5,6]. There is a need for further team-based working approaches in health care, with a focus on continuity of care [4,7,8]. This knowledge has led to the implementation of various tailored PHC programmes in numerous countries [8–11]. In interview studies, PHC professionals have suggested that proactive, integrated care may improve quality of care for older patients [4,12]. Lack of geriatric competence, economic restrictions and time constraints have been reported as barriers to such an approach [7,13].

In the few previous studies where older patients’ perspectives have been explored, it has come to light that they have a more passive role in medical decisions than they would like. Many wish to take part in the decisions regarding their care; however, to facilitate patient participation, health care professionals need to take the initiative and invite the older patients to take part [14,15]. Involving the patient in medical decisions has been associated with improved treatment outcomes and patient satisfaction [16] and is a part of the World Health Organization (WHO) recommendations [17]. Two potential barriers for such involvement are a lack of professional support and poorly adapted health care models. Hence, there is an increased need for interventions and support that are based on older patients’ needs [7,18].

Older people with chronic conditions describe their experiences with health care as fragmentised; common experiences are lack of continuity and an impression of slipping through the net [15,19]. This particularly applies to people with multiple morbidity, complex needs and inferior resources, and is among other things caused by lack of communication and lack of coordination within health care. An important factor in preventing these feelings of slipping through the net is the opportunity to see a known and trusted health care professional [19]. Insufficient information and unclear distribution of responsibility may hinder that continuity [3,7,9,15,18].

Numerous health care centres for older patients and geriatric outpatient teams have been initiated in Sweden to improve the standard of care. However, there are few scientific evaluations of those interventions [20–22]. In a Swedish randomised controlled study by Ekdahl and colleagues [20], the effect of a geriatric outpatient team was compared with ordinary care. After three years, the participants in the intervention group showed an increased lifespan, fewer care days in hospital, with no increased cost compared to ordinary care. The key factors in the success of the method were identified as access to staff with geriatric competence, interprofessional teamwork, cooperation and more patient contact time for health care staff [20,21].

Older patients are a marginalised group whose voice is important to listen to in order to achieve person-centred care. How we can design this person-centred care in accordance with the individual needs of older patients is an important research question [3,8,22]. Moreover, the issue of how to increase the participation of older patients in their own care needs to be examined further [6,23]. Providing tailored care to older patients in PHC has shown promising results regarding quality of care [4,5,12]; however, a deeper exploration of older patients’ own perspective is needed to further develop their care in PHC.

The aim of this study was to explore older patients’ experiences of a tailored PHC unit.

## Material and methods

A qualitative explorative study design was chosen and data were collected through semi-structured interviews. An explorative design was chosen because it can provide knowledge in a research area that has been sparsely investigated [3,6,10,18,24]. Individual interviews were held to provide a rich understanding of older patients’ experiences of a tailored PHC unit [25]. The interview transcripts were analysed inductively using qualitative content analysis [25].

### Setting

The responsibility for health and medical services in Sweden lies within the county councils and regions and is funded almost entirely by taxes. Municipalities provide social services and certain health care services, such as nursing homes, palliative care and home health care. General health care services are provided by PHC; this means that older people have to encounter two different institutions when in need of both medical and social care. Service provision varies widely across the 20 county councils and regions and the 290 municipalities, due to their high degree of political autonomy [26]. However, awareness has grown in the last decade that there has to be an increased cooperation between the different organisations that provide care for older people in order to meet their complex needs [21]. In December 2017, a unit for people aged 75 years or older was set up in a PHC centre in a region in central Sweden in order to improve care for older people. The environment at the unit was designed to facilitate accessibility, and there was a focus on inter-professional collaboration. Staffing level on a daily basis were two physicians, two district nurses, one physiotherapist and one nursing assistant working in teams. The appointments were longer than ordinary primary care appointments. There was a focus on obtaining health or preventing declining health in older patients, using a structured approach. The structured approach focused on the prevention of falls, malnutrition and decubitus ulcers, along with the detection of dementia and the assessment of medications. Furthermore, the district nurses working at the unit had a specific role to coordinate health care with municipality care.

### Participants

A purposeful sampling strategy was used. Older patients who had visited the tailored PHC unit were eligible to participate in the study. Participants were recruited by nurses when they contacted or visited the PHC unit. The inclusion criteria were being 75 years of age or older, having at least two chronic diagnoses, having visited the tailored PHC unit at least five times in the past year and being Swedish speaking. The exclusion criterion was having a severe psychiatric or neurological condition with severe cognitive disabilities, but patients with minor cognitive disabilities only could be included. Patients who were or had been in a care relationship with the interviewer (first author, UW), who has a nursing background, were excluded in order to enable participants to speak freely about both positive and negative experiences. Those who met the inclusion criteria were given brief oral and written information about the study by the nurse. Written information was also available in the PHC centre in the form of posters and leaflets.

The purposeful sampling strategy was used to achieve a heterogeneous sample of participants based on age and gender in order to obtain the richest possible data [25].

Patients interested in participating were contacted by one of the authors (UW) for further verbal and written information about the study and were given a few days to consider participation. Then they were contacted again and a time and place for the interview was agreed upon with those who gave consent to participate. One author (UW) conducted the interviews with five men and 14 women in February to September 2019. After 19 interviews and repeated evaluation of the recordings, the researchers found that there was sufficient information power. Therefore, the number of participants was deemed to be sufficient [27]. The characteristics of the participants are shown in Table 1.

**Table 1.** Patients’ characteristics.

Characteristics	Total (*n*)
Gender	
Male	6
Female	13
Age groups (years)	
80–84	5
85–89	10
90–94	4
Chronic diseases	
2	10
3–5	7
6 or more	2
Visits (unit for eldely)	
5	7
6 or more	11
Unknown	1
Marital status	
Singel	12
Married	7
Domestic care	
Yes	4
No	14
Occasional	1
Use of assistive devices	
None	14
Walker outdoors	3
Walker indoors and outdoors	1
Wheelchair	1
Level of education	
Elementary school < 9 years	13
Elementary school 9 years	1
College	3
University	2

### Data collection

A semi-structured interview guide was developed by the authors, based on previous studies, discussions within the research group and presentations at a scientific seminar. Three pilot interviews were conducted in order for the first author (UW) to further refine the interview technique and to evaluate the questions. As a result of these interviews, one question was added to the interview guide: ‘When seeking care, what do you want a primary health care unit for older patients to do for you?’ which gave a more descriptive response. Furthermore, follow-up questions were added to deepen the responses [25]. The pilot interviews were not included in the final dataset.

The final interview guide contained open-ended questions regarding older patients’ experiences of a PHC unit for older patients (Appendix 1). The interviews took place at participants’ homes or the PHC centre, and one was conducted by phone, according to the participants’ wishes. Prior to the interview, written informed consent and additional background data were collected. The interviews were audio-recorded and each interview lasted between 10 and 49 min, with a median of 24 min. The initial four interviews were transcribed verbatim by the author (UW), and the remaining verbatim transcriptions were provided by a professional transcriber. After each interview, the material was checked by listening to the audio file and reading the transcription. The transcribed interviews were imported into the software program NVivo 12 (QSR International, Melbourne, Australia) in order to manage and code the data [28]. Each participant was pseudonymised and given a number from 1 to 19.

### Data analysis

Data were analysed using qualitative content analysis with an inductive approach [25] to identify the underlying structure of older patients’ experiences of a tailored PHC unit. The data analysis began when all 19 interviews were conducted. Regular discussions were held during the interviewing period between the first author (UW) and an experienced qualitative researcher (ENS) to get an understanding of the aggregated data. Initially, the transcriptions were read through several times to become familiar with the material. Meaning units that referred to the aim which was to describe older patients’ experiences of a tailored PHC unit were highlighted and then condensed into codes. The authors (UW and ENS) discussed back and forth the emerging codes, subcategories and categories, which were compared for similarities and differences. Furthermore, the emerging subcategories, categories and quotes were discussed by analytical triangulation [25] in the research group at repeated sessions. Finally, all authors discussed the categorisation and agreed upon the final version. Example of the analysis process is provided in Table 2. The research group consisted of an occupational therapist (LH), a physician (MH), a physiotherapist (ENS) and a district nurse (UW), all with professional experience of care for older patients in PHC. One author (UW) had experience from working at the tailored PHC unit.

**Table 2.** Example of the analysis process.

Quotes	Coding	Subcategory	Category	Theme
Because you get there and you feel the tranquility, no urgency, nothing. Like the first time I was there for an hour, but goodness me, this can't be true!	The importance of having enough time when in contact with the unit	Structural factors promoting easy access and continuity of care.	Feeling secure as a patient	In safe hands when in need of primary health care
… it’s not loads of people running around like ants and white coats and it feels stressful, rather it's a bit relaxed environment, I would say, you get that feeling, yes … I experience it. And that’s good…	The importance of the environment for older people's experience of care			
I have a treatment (laughs) right now that is not very nice but but it will be anyway because they are very nice staff, it makes a big difference.	Older people feel that they are treated kindly by the staff	PHC professionals’ positive approach		
I understand that you have a lot to do, I think when I come I will be taken care of so terribly, it’s fantastic	Older people feel that they are taken care of when in contact with the unit			

### Ethical approval

The study was approved by the Regional Ethical Review Board in Uppsala, approval number 2017/516, and followed the principles defined in the Declaration of Helsinki [29]. The audio files and transcriptions were coded and saved on a password-protected server. The code key and other relevant material is stored in a safe locker accessible only to authorised personnel. Only coded material was discussed among the researchers. During a few interviews, health issues that required further attention came to light and those participants received contact with appropriate PHC professionals for help.

## Results

In the analysis, the theme *In safe hands when in need of primary health care* emerged. The theme consists of three categories and seven subcategories, which are presented in Table 3. The findings are illustrated with quotations from the participants, and Table 2 shows an example of the analysis process, deriving codes, subcategories and categories from the quotations.

**Table 3.** Overview of categories and subcategories describing older patients’ experiences of a tailored primary health care unit.

Theme	Category	Subcategory
In safe hands when in need of primary health care	To receive the needed care	Preventive activities to identify health issues in time
		Multiprofessional competence to meet older people’s individual needs
		Support and actions from PHC professionals to meet individual needs
	To feel secure	Structural factors promoting interaction and safety
		The PHC professionals positive approach
	To be seen and participate in care	To build a mutual partnership
		To be seen as a unique person /an individual with personal needs

### Theme: in safe hands when in need of primary health care

The tailored PHC unit was highly valued by the participants. Both structural and professional factors facilitated a more person-centred care and enabled the older patients to participate in their care. This unit provided care tailored to older patients’ needs and was perceived as an improvement compared with ordinary PHC centres. The participants felt that they were in safe hands, and they emphasised that it was the best thing that has happened in PHC.

#### Category: being seen and participating in care

This category emerged from two subcategories which refer to the participants’ experiences of being seen by and interacting with PHC professionals. The category indicates that the participants wanted to participate in their care, which was facilitated when receiving understandable information and feeling listened to by a likeable PHC professional.

#### Subcategory: being seen as a unique person with individual needs

The participants talked about the importance of being asked about their thoughts and feelings when in need of care. They commented that their issues were taken seriously and they felt listened to when in contact with the professionals at the tailored PHC unit. They further reported that they were not treated just as an older patient but as an individual and they stressed how important this was for them. They appreciated feeling acknowledged as a person even though they were severely ill and had many problems.

I can say that this is how you want to be treated, they believe what I say and have ideas about what the causes may be … Yes, that’s how I feel that it works very well. (P5)

The participants reported that their need for information was met at the tailored PHC unit. The information was understandable, with clear instructions in order to avoid misunderstandings. They further commented that it was important for them to understand their situation to avoid guesswork and misconceptions about their conditions. They felt that PHC professionals should make sure that the older patient has perceived the information correctly before leaving the consultation.

And then it’s good if the person is good at explaining, as XX has done, she has explained and put in words illustrating one thing and another.(P18)

#### Subcategory: building a mutual partnership

The participants stressed that they wanted to participate in their care and took the initiative by interacting with PHC professionals at the tailored unit. They asked questions about their condition in order to understand how it affected them and they described their particular circumstances in order to receive the care they needed. Moreover, they talked about the importance of liking the professionals who were going to care for them and having a feeling of getting along. The participants commented that they dared to speak during health care consultations if they felt an acquaintance with the PHC professionals.

Yes, you get a trust … between us, huh, I think… you know what to expect of each other pretty much (laughs). It is worth a lot. (P19)

The participants pointed out that they were not afraid of saying something stupid and that they could discuss everything. They expressed feelings of being a team with the PHC professionals at the tailored unit, which they had not experienced at the ordinary PHC centre.

And not just sitting and waiting, as now the doctor will talk and inform, because I can also talk. You could never do that before, no. (P10)

#### Category: feeling secure as a patient

This category concerned structural and professional factors that affected the likelihood that the participants would feel secure through their contact with the tailored PHC unit. The category revealed that easy access, enough contact time and a calm environment, along with the PHC professionals’ approach, increased the participants’ ability to feel secure.

#### Subcategory: structural factors promoting easy access and continuity of care

The tailored PHC unit offered easy contact, which was highlighted by the participants as important for them to feel confident that they would receive the care they needed.

And I know when I go down there, I get help right away, it’s been great. (P16)

Furthermore, the participants raised the issue of having contact with the same PHC professionals. They felt that repeatedly seeing the same person facilitated a close patient–professional relationship and was reassuring, making them feel safe and confident.

I’m not afraid as I was before, because then I got so many different doctors up there. (P2)

Additionally, the participants emphasised time as very important. When given enough time during health care consultations, they felt calm and had the opportunity to talk thoroughly about their health problems. Their experience was that the PHC professionals at the tailored unit had enough time to listen to them, which contributed to feelings of security.

They described the environment at the tailored PHC unit as calm and relaxing, which they found helpful when they did not feel well. The atmosphere contributed to feelings of not being ill and enabled the participants to relax before health care visits. They commented that this was a striking difference compared to the ordinary PHC center, where there was a much more stressful atmosphere.

You go here because it’s nice. It’s such a significant difference, so it’s beyond comparison. (P11)

#### Subcategory: PHC professionals’ positive approach

The PHC professionals’ approach was described by the participants as welcoming. This kind of welcoming gave them a feeling of being important and that it was pleasant for them to attend a unit that was just for them. They stated that they did not have to worry because they knew that the PHC professionals were there for them. They felt trust that they were taken seriously and could bring up anything they wanted to discuss, no matter how big or small.

‘Yes, but you feel that … that they care … that’s probably what I call trust.’ (P8) The participants further reported that they perceived the PHC professionals at the tailored unit as friendly, which also contributed to their feeling that they would be taken care of. There were descriptions of unpleasant treatments that were easier to bear when administered by a nice PHC professional. Furthermore, the participants felt that everyone was willing to help them and that nothing was impossible.

There is nothing that is impossible there, so to speak. On the contrary, they are all willing to help, that’s what’s so nice, both doctors and nurses and head nurses and everything. (P2)

#### Category: receiving the needed care

This category consists of three subcategories revealing that PHC professionals were perceived to have geriatric competence and to take responsibility for the care of older patients. Even though the participants experienced that all their health problems were given full attention, it emerged that the focus on preventive care was limited.

#### Subcategory: support and actions from the PHC professionals to meet individual needs

The participants observed that professionals at the tailored PHC unit were thorough during consultations and looked everything up. If there were any uncertainties, they searched for answers to be able to establish the right treatment; they would consider everything and then talk it through with the patient. Everything was attended to during visits and the participants said they never had to remind the PHC professionals of what needed to be done. The tailored PHC unit never abandoned its responsibility of care, which was a common experience with the ordinary PHC centre. The participants reported that they were offered follow-up appointments to make sure everything worked out well for them, and these actions made them feel supported and well taken care of.

He is great, he has called regularly, when I went to the ordinary health centre then you barely got, then you had to get up and ask for the answers sometimes, no, so it is a huge difference, it is. (P2)

#### Subcategory: interprofessional competence to meet older people’s individual needs

The participants highlighted the importance of being taken care of, in terms of being acknowledged, understood, thoroughly examined and receiving a diagnosis and proper treatment according to their specific needs. They perceived that the professionals at the tailored PHC unit as having specialist knowledge about age-related health problems, which they described as reassuring. Just knowing that the tailored PHC unit was focusing on older patients gave the participants confidence that they were going to be taken care of.

In cases where health issues could not be handled at the tailored unit for older patients, the PHC professionals referred the patient to specialist care in order to help them. The participants stressed that this directly catered to their individual needs.

Despite the participants’ experience that they were being taken care of by professionals with geriatric competence, they expressed a further need for PHC units for older patients to address social needs as well as medical needs. They needed to know where and whom to turn to in the municipality when in need of domestic help. Some wished for a contact network to be incorporated at the tailored PHC unit in order to facilitate such contact. Others wanted help to be connected with people in a similar situation, to reduce loneliness.

‘And then they can ask “Where do you live?” yes then you live on the 3rd floor without an elevator. “Yes, but then we have someone that we can contact here at the municipality about where there are opportunities for you to join the queue or look for another home that suits you, where you at least have an elevator or something.” Then you can have … take it in connection with everything else … And then this social thing with what there is, it is, I think, you should connect them up, but then there must be resources, people who, who the doctor can then give tips to and you will get appointments and help and where to go. Many old people may not know where to go?’ (P17)

#### Subcategory: preventive activities to identify health issues in time

The participants reported that, when visiting the tailored PHC unit, they felt well taken care of and had the opportunity to review their medications, get instructions about a preferable diet and appropriate physical activities; however, they expressed a need for preventive health check-ups. They said that they did not seek care when experiencing minor health issues and that it would be preferable to receive an invitation to a preventive health check-up to detect and target health-related problems before they develop into sickness. They thought that preventive care was hindered by the financial constraints in the region, but it would be favourable for both older persons and the health care system. They suggested that there should be a dietician connected to the unit for nutritional advice, and some also wanted compulsory physical exercise. They pointed out that they needed instructions and help to establish a routine with such activities, and if these were not compulsory it would not be much exercise done at all.

and that there will not be so many old people who will get sick, so we will prevent instead of taking care of it when it is already too late (P17).

## Discussion

This study is, to our knowledge, the first to explore the experiences of older patients regarding tailored PHC units in Sweden. Prior studies have shown that there is an increased need for tailored interventions based on older patients’ needs in order to further refine PHC [7,18]. According to WHO [17], such interventions are needed in order to improve the care of older people. In Sweden, various PHC interventions have been set up in order to enhance the care of older patients, but there is a lack of scientific evaluations to confirm whether these interventions actually meet older patients’ needs.

### Principal findings

The older patients who were interviewed in our study were highly appreciative of the tailored PHC unit they attended and emphasised that it was an improvement in care compared to ordinary PHC centres. The theme that emerged in our analysis of the interview transcripts was that the participants experienced the unit as a place where they were in safe hands when in need of primary health care; moreover, they described it as the best thing that has happened for older patients in PHC. Previous findings by Vestjens [30] have shown that a productive patient–professional interaction can contribute to older patients’ wellbeing and prevent decline in their health. In order to reach that productive interaction, relevant expertise, time and patient information are needed. Increased patient contact time at PHC centre visits has been associated with higher patient satisfaction [21], which is confirmed by the participants in our study; they emphasised the importance of being allowed enough time to build a partnership with PHC professionals. According to Kelly [7], time constrains are a major obstacle to patient-centred communication and an innovative PHC would improve the wellbeing of older patients. In an evaluation of a care approach for healthy ageing in Europe [5], trust appears to be the foundation of the partnership between older patients and care providers. The participants in our study confirmed that a friendly PHC professional made them feel safe. They highlighted continuity, easy access and a calm environment as important differences compared to the ordinary PHC centre. Furthermore, having PHC professionals who cared about them and attended to their individual needs was something they had not experienced at the ordinary PHC centre.

The participants reported that they were able to participate in their care at the tailored PHC unit. Previous studies concluded that, if professionals give insufficient support and do not involve older patients and consider their special needs, these patients will have difficulties to participate in their care [7,18].

Another neglected issue is how health services should adapt in order to invite older patients to participate in designing tailored care [6]. A study in the Netherlands by Hoogendijk [11] evaluating integrated care programmes showed that these programmes have limited evidence for effectiveness; however, they tend to focus on health care costs and not perceived quality of care. Hence, King et al. [31] reported that an intervention with gerontology nurse specialists in primary care was highly regarded by both older patients and professionals. King *et al.* argued that individual value is rarely acknowledged but should be seen as a vital component in the care of older patients.

The participants in our study expressed a wish for preventive health check-ups and activities, although they believed that financial constraints would be an issue. Bleijenberg [13] argued that the health care sector needs financial compensation in order to be able to provide proactive care to older patients. The participants in our study talked about the importance of integrating social services at the PHC unit. Indeed, other studies [3,12,32,33] have concluded that an integrated care approach tailored to older patients’ needs is necessary for a more comprehensive geriatric primary care model. According to Briggs [10], interventions that focus on integrating health and social care are needed to better understand person-centred outcomes and experiences of care. This is in line with the suggestion by WHO [17] that services need to be coordinated according to older people’s preferences, needs and goals.

This study has shown that older patients have a need for a tailored PHC environment with generous contact time in order to feel safe in the knowledge that their needs will be met. They also need preventive health check-ups and an enhanced coordination between social and health care.

PHC centres should strive to create a calm environment, secure geriatric competence and enable enough contact time with older patients to be able to meet their needs. Furthermore, to improve wellbeing, PHC centres should incorporate social services along with preventive health check-ups for older patients.

Future studies ought to include informal carers, since they play a crucial part in many older patients’ lives [3,8,12] and understand the person’s individual needs.

Our study has shown that older patients greatly valued having a tailored PHC unit. Whether such units are also cost-effective is still unknown, and this ought to be established as the next evaluation step. It would be preferable to combine an evaluation of patient-reported outcomes and cost-effectiveness to reach a holistic understanding about the care of older patients in PHC.

### Strengths and weaknesses of the study

This qualitative study has both strengths and limitations that needs to be considered when interpreting the findings. We employed a qualitative study design based on individual interviews with open-ended questions, allowing participants to express their experiences [25]. The knowledge that the interviewer was a health care professional may have restrained the participants from speaking freely about the tailored PHC unit. To counteract this, the participants were able to choose the place for the interview and efforts were made to create a relaxed and permissive atmosphere. The participants expressed ideas of improvement of the tailored PHC unit which indicates that they felt free to speak during the interviews despite knowledge of the interviewers (UW) occupation. The data were analysed systematically and close to the text by using qualitative content analysis [25]. To strengthen confirmability, continuous discussions were held throughout the analysis process, and the entire research group approved the findings. The research group consisted of a district nurse (UW), a physiotherapist (ENS), an occupational therapist (LH) and a physician (MH). This multidisciplinary research group has good scientific knowledge and clinical experience of treating older patients in PHC. The preunderstanding of PHC may have affected the data collection, analysis and results. The first author (UW) had working experience from the tailored PHC unit with an awareness of the importance of improving the care of older patients in PHC. However, these perspectives were considered throughout the performance of the study and continuous discussions were held between the authors and the final categories were discussed at a seminar in a larger research environment to enhance trustworthiness. The interview guide was pilot-tested and none of the participants had a caring relationship with the researchers, which strengthens credibility. Neither transcripts or findings were returned to participants for comments.

In this qualitative research there are no claims of generalisability, although the findings may be transferable to similar settings.

### Conclusion and implications for policy and practice

A tailored PHC unit for older patients was appreciated by the study participants in the target group. They described it as a place where they could be in safe hands when in need of primary health care, and they emphasised that it was an improvement in care compared to ordinary PHC centres. The results provide insights into older patients’ experiences, which can be helpful in the ongoing process of improving the care of older persons in PHC.

## Appendix 1

**Interview guide**

1. When visiting the primary health care unit for older patients, what is important for you?
2. Can you tell me about your last visit to the primary health care unit for older patients?
3. Can you tell me about a visit that has worked out well for you?
4. Can you tell me about a visit that has worked out less well for you?

Follow-up questions if the answers did not emerge spontaneously:

What was the reason for the visit? Who did you meet? What happened?
5.When seeking care, what do you want a primary health care unit for older patients to do for you?
6.Is there anything more you want to tell me that we have not talked about already?

Follow-up questions:

Do you want to describe more closely…?
Can you tell more about…?
How did you experience that?
How do you mean?

## References

[1] Amalberti R, Vincent C, Nicklin W, et al. Coping with more people with more illness. Part 1: the nature of the challenge and the implications for safety and quality. Int J Qual Health Care. 2019;31(2):154–158. 30476145 10.1093/intqhc/mzy235

[2] Lim WS, Wong SF, Leong I, et al. Forging a frailty-ready healthcare system to meet population ageing. Int J Environ Res Public Health. 2017;14(12):1448. 10.3390/ijerph14121448 PMC5750867 29186782

[3] McGilton KS, Vellani S, Yeung L, et al. Identifying and understanding the health and social care needs of older adults with multiple chronic conditions and their caregivers: a scoping review. BMC Geriatr. 2018;18(1):231. 30285641 10.1186/s12877-018-0925-x PMC6167839

[4] Vestjens L, Cramm JM, Nieboer AP. An integrated primary care approach for frail community-dwelling older persons: a step forward in improving the quality of care. BMC Health Serv Res. 2018;18(1):28. 29343253 10.1186/s12913-017-2827-6 PMC5773125

[5] Franse CB, Zhang X, van Grieken A, et al. A coordinated preventive care approach for healthy ageing in five European cities: a mixed methods study of process evaluation components. J Adv Nurs. 2019;75(12):3689–3701. 31441529 10.1111/jan.14181

[6] Fusco F, Marsilio M, Guglielmetti C. Co-production in health policy and management: a comprehensive bibliometric review. BMC Health Serv Res. 2020;20(1):504. 32503522 10.1186/s12913-020-05241-2 PMC7275357

[7] Kelly G, Mrengqwa L, Geffen L. “They don’t care about us”: older people’s experiences of primary healthcare in Cape Town, South Africa. BMC Geriatr. 2019;19(1):98. 10.1186/s12877-019-1116-0 PMC6449977 30947709

[8] Valaitis R, Cleghorn L, Dolovich L, et al. Examining Interprofessional teams structures and processes in the implementation of a primary care intervention (Health TAPESTRY) for older adults using normalization process theory. BMC Fam Pract. 2020;21(1):63. 32295524 10.1186/s12875-020-01131-y PMC7160930

[9] Bleijenberg N, Boeije HR, Onderwater AT, et al. Frail older adults’ experiences with a proactive, nurse-led primary care program: a qualitative study. J Gerontol Nurs. 2015;41(9):20–29. quiz 30-1. 26375146 10.3928/00989134-20150814-03

[10] Briggs AM, Valentijn PP, Thiyagarajan JA, et al. Elements of integrated care approaches for older people: a review of reviews. BMJ Open. 2018;8(4):e021194. 10.1136/bmjopen-2017-021194 PMC5892746 29627819

[11] Hoogendijk EO. How effective is integrated care for community-dwelling frail older people? The case of The Netherlands. Age Ageing. 2016;45(5):585–588. 27146300 10.1093/ageing/afw081

[12] Ploeg J, Yous ML, Fraser K, et al. Healthcare providers’ experiences in supporting community-living older adults to manage multiple chronic conditions: a qualitative study. BMC Geriatr. 2019;19(1):316. 31744477 10.1186/s12877-019-1345-2 PMC6862842

[13] Bleijenberg N, Ten Dam VH, Steunenberg B, et al. Exploring the expectations, needs and experiences of general practitioners and nurses towards a proactive and structured care programme for frail older patients: a mixed-methods study. J Adv Nurs. 2013;69(10):2262–2273. 23461433 10.1111/jan.12110

[14] Wissendorff Ekdahl A. Frail and elderly hospital patients: the challenge of participation in medical decision making [doctoral thesis, comprehensive summary]. Linköping: Linköping University Electronic Press; 2012.

[15] Rustad EC, Furnes B, Cronfalk BS, et al. Older patients’ experiences during care transition. Patient Prefer Adherence. 2016;10:769–779. 27274204 10.2147/PPA.S97570 PMC4869594

[16] Kaplan RM, Frosch DL. Decision making in medicine and health care. Annu Rev Clin Psychol. 2005;1:525–556. 17716098 10.1146/annurev.clinpsy.1.102803.144118

[17] Araujo de Carvalho I, Epping-Jordan J, Pot AM, et al. Organizing integrated health-care services to meet older people's needs. Bull World Health Organ. 2017;95(11):756–763. 29147056 10.2471/BLT.16.187617 PMC5677611

[18] Abdi S, Spann A, Borilovic J, et al. Understanding the care and support needs of older people: a scoping review and categorisation using the WHO international classification of functioning, disability and health framework (ICF). BMC Geriatr. 2019;19(1):195. 31331279 10.1186/s12877-019-1189-9 PMC6647108

[19] Tarrant C, Windridge K, Baker R, et al. Falling through gaps’: primary care patients’ accounts of breakdowns in experienced continuity of care. Fam Pract. 2015;32(1):82–87. 25411422 10.1093/fampra/cmu077 PMC5926435

[20] Ekdahl AW, Alwin J, Eckerblad J, et al. Long-term evaluation of the Ambulatory Geriatric Assessment: a Frailty Intervention Trial (AGe-FIT): clinical outcomes and total costs after 36 months. J Am Med Dir Assoc. 2016;17(3):263–268. 26805750 10.1016/j.jamda.2015.12.008

[21] Ljungbeck B, Sjögren Forss K. Advanced nurse practitioners in municipal healthcare as a way to meet the growing healthcare needs of the frail elderly: a qualitative interview study with managers, doctors and specialist nurses. BMC Nurs. 2017;16(1):9. 29176932 10.1186/s12912-017-0258-7 PMC5689167

[22] Stoop A, Lette M, Ambugo EA, et al. Improving person-centredness in integrated care for older people: experiences from thirteen integrated care sites in Europe. Int J Integr Care. 2020;20(2):16. 10.5334/ijic.5427 PMC7319083 32607103

[23] Malterud K, Kamps H. General practice - a fertile lagoon in the ocean of medical knowledge. Scand J Prim Health Care. 2021;39(4):515–518. 34783285 10.1080/02813432.2021.2004831 PMC8725917

[24] Shepherd V, Wood F, Hood K. Establishing a set of research priorities in care homes for older people in the UK: a modified Delphi consensus study with care home staff. Age Ageing. 2017;46(2):284–290. 27852597 10.1093/ageing/afw204 PMC5386006

[25] Patton MQ. Qualitative research & evaluation methods. London: SAGE; 2002.

[26] Johansson L, Long H, Parker MG. Informal caregiving for elders in Sweden: an analysis of current policy developments. J Aging Soc Policy. 2011;23(4):335–353. 21985063 10.1080/08959420.2011.605630

[27] Malterud K, Siersma VD, Guassora AD. Sample size in qualitative interview studies: guided by information power. Qual Health Res. 2016;26(13):1753–1760. 26613970 10.1177/1049732315617444

[28] Jackson K, Bazeley P. Qualitaive data analysis with Nvivo. London: SAGE; 2019.

[29] World Medical Association. 2018. Declaration of Helsinki: ethical principles for medical research involving human subjects. https://www.wma.net/policies-post/wma-declaration-of-helsinki-ethical-principles-for-medical-research-involving-human-subjects/. 19886379

[30] Vestjens L, Cramm JM, Nieboer AP. A cross-sectional study investigating the relationships between self-management abilities, productive patient-professional interactions, and well-being of community-dwelling frail older people. Eur J Ageing. 2021;18(3):427–437. 34483806 10.1007/s10433-020-00586-3 PMC8377131

[31] King AII, Boyd ML, Dagley L, et al. Implementation of a gerontology nurse specialist role in primary health care: health professional and older adult perspectives. J Clin Nurs. 2018;27(3-4):807–818. 29052288 10.1111/jocn.14110

[32] Metzelthin SF, Daniels R, Rossum E, et al. A nurse-led interdisciplinary primary care approach to prevent disability among community-dwelling frail older people: a large-scale process evaluation. Int J Nurs Stud. 2013;50(9):1184–1196. 23384696 10.1016/j.ijnurstu.2012.12.016

[33] Nord M, Östgren CJ, Marcusson J, et al. Staff experiences of a new tool for comprehensive geriatric assessment in primary care (PASTEL): a focus group study. Scand J Prim Health Care. 2020;38(2):132–145. 32349567 10.1080/02813432.2020.1755786 PMC8570711

